# Physicians’ use of pain scale and treatment procedures among children and youth in emergency primary care - a cross sectional study

**DOI:** 10.1186/s12873-015-0059-9

**Published:** 2015-11-06

**Authors:** Svein-Denis Moutte, Christina Brudvik, Tone Morken

**Affiliations:** National Centre for Emergency Primary Health Care, Uni Research Health, Bergen, Norway; Bergen Accident and Emergency Department, Bergen, Norway; Department of Clinical Medicine, University of Bergen, Bergen, Norway; Department of Global Public Health and Primary Care, University of Bergen, Bergen, Norway

**Keywords:** Pain assessment, Child, Youth, Accident and emergency department, Primary care physician

## Abstract

**Background:**

Pain is a common symptom in children and youth attending casualty centres and emergency departments. The aim of this study was to acquire more knowledge about how pain in children is measured and handled by emergency primary care physicians.

**Methods:**

A structured questionnaire study was performed among 75 emergency primary care physicians in a Norwegian accident and emergency department (AED). We used descriptive statistics to analyse the use of a pain scale, the use of weight and age when dosing pain medication, the need for more knowledge and the need for pain management procedures in children. The Pearson chi-square test was used to analyse differences between groups.

**Results:**

A pain scale with a visual analogue scale (VAS) had been used by 59 % of physicians in young patients aged 9 to 19 years, by 23 % in children aged 3 to 8 years, and by 3 % in children below 3 years. A total of 63 % of physicians reported that they used the child’s weight instead of the age interval when estimating the needed dose of painkillers. They relied on parents’ weight estimation and seldom measured the child’s weight at attendance. Most emergency medical care physicians reported a need for more knowledge and better procedures related to both pain evaluation and pain treatment in children and youth. The physicians included in the study were demographically representative of AED physicians in Norway (average age 37 years old, 55 % men, 76 % had studied medicine in Norway and 49 % had fewer than 5 years of medical experience).

**Conclusions:**

Emergency primary care physicians report a need for pain assessment procedures in children and youth. They sometimes use a pain scale when measuring and managing pain in patients aged 9 to 19 years, but seldom in younger patients.

## Background

Pain is the most common symptom in children and youth attending casualty centres and emergency departments whether due to middle ear infections, appendicitis, fractures or wounds [1, 2]. To make a correct diagnosis, it is important for the physician to evaluate the quality, quantity, location, affective and cognitive aspects and other characteristics related to the pain. Pain evaluation is more difficult the younger and less verbally communicative the child is. We also know that visceral and chronic pain is more difficult to evaluate than somatic and acute pain [2]. Inadequate pain management during medical care, especially among very young children, can have detrimental effects [3].

### Pain relief procedures

During the recent years, increasing international attention has been focused on children with pain, and various improvements in pain relief have been introduced [4–6]. Despite this, there is still recognized common consensus that children’s pain is underestimated and undertreated [1, 2]. Many Norwegian hospitals have no specific routines or procedures for how to manage pain in children [7]. In North America, pain treatment of children at emergency clinics has been found to be inadequate compared to pain treatment of adults with similar medical conditions [4, 8]. According to the recommendations of The Joint Commission [9], all patients should receive an adequate evaluation and treatment of their pain, independent of the underlying medical condition. This implies early evaluation, continuous re-evaluations, documentation and reporting of pain [10].

### Pain scale

The usefulness of pain intensity scores in a busy pre-hospital setting is still an item of controversy among clinicians. It is a simplification to describe pain in terms of its intensity alone, but it nevertheless seems useful in evaluating both the effects of pain-relieving and pain-producing interventions [10]. The registration of variations in pain intensity is also important in the diagnostic process itself. A graded scale is necessary because pain treatment may be effective even without eliminating all pain. Many studies have shown that pain management is improved when pain is regularly and reliably measured [6, 9–11]. Pain scales were developed more than 20 years ago, and the most commonly used scales have been validated in different age groups [12–14]. No single scale seems to be optimal for use with all types of pain or across the developmental age span, and age-based recommendations have been developed [15]. As pain scales may not be interchangeable, it is important that the same type of pain scale is used both before and after the pain relieving intervention [13].

### Dosage and overweight

Many mistakes are made in the medication of children. Errors occur when prescribing, dispensing and administering medication, especially when the use of analgesics, sedatives and antibiotics [16, 17] is involved. In France, it is recommended that the physician notes the child’s age and weight when prescribing medication for children aged 0–14 years to avoid dosing errors [18]. Weight will often be the most important parameter for determining the correct drug dose for children older than six months of age. In recent years, a higher proportion of children in the western world, including Norway, have been classified as obese [19]. Dosing by age alone increases the risk of being insufficiently medicated, especially in an accident and emergency department (AED) setting where sufficient analgesia is important before painful procedures. In an acute situation and in a short course of treatment, this is hardly a problem. On the other hand, it has been found that physicians, nurses and parents who try to estimate weight often give a lower weight than the actual weight of the child [20]. This can also lead to an insufficiently low dosage of medications. In the Norwegian medical formulary, Felleskatalogen, drugs are commonly dosed by age groups.

The aim of this study was to acquire more knowledge about how pain in children is measured and handled by physicians working in a larger Norwegian casualty centre.

## Methods

This cross sectional study was conducted at Bergen Accident and Emergency Department (AED), a large combined emergency primary care centre and emergency department. Most patients coming from the population in the greater Bergen area receive treatment here, representing some 100 000 consultations of which 19 000 are adolescents and children under 20 years of age. Bergen AED has an injury division that treats patients with different types of injuries including fractures, dislocations, contusions and wounds. The general medicine division receives patients with many different conditions involving pain like middle ear infections and abdominal pain. During daytime, Bergen AED is mostly staffed by physicians who are permanent employees or interns, but during evenings and nights, most physicians on call are self-employed general practitioners (GPs) or hospital physicians.

During a period of 17 days in November 2011, all physicians working at Bergen AED were invited to participate in an anonymous paper survey. The project leader (first author and physician at the Bergen AED) distributed an individual questionnaire in Norwegian to every participating physician. Personal data regarding age, gender, parental status, country of birth and professional experience were recorded. Following this, the physicians answered standardized and graded questions about pain assessment in children. They were asked how difficult they found it to evaluate pain in children, if and how often they used pain scales and which method or methods they used to calculate drug doses. Further questions involved whether they felt they needed more knowledge about pain management in children, whether they wanted to receive specific procedures for assessment and treatment of pain in children, and whether they felt a need for alternative options regarding analgesics (oral soluble tablets, tablet size, taste and the like) than those currently available to children in Norway. Finally, they were asked to assess how satisfied they were with their daily management of children with pain. Their responses were graded according to a five-point scale with the options ‘not at all’ , ‘to a small extent’ , ‘to some extent’ , ‘largely’ and ‘to a very large extent’.

The IBM-SPSS version 19 was used for statistical analysis. The level of statistical significance was set at 5 % (*p* < 0.05). We used Pearson chi-square test in cross tabulation for nominal values to test differences between groups.

The Norwegian Ethical Committee for Medical Research approved the study.

## Results

The participants in this study were 75 of 76 physicians working at Bergen AED. On average, they were 37 years old, 55 % were men, and 52 % had children of their own [Table 1]. There was no significant difference between female and male physicians with respect to age, number of children and professional experience. A total of 76 % were born in Norway, 9 % in a European country other than Norway and 15 % outside Europe. Most of the physicians (73 %) had studied medicine in Norway; 24 % in another European country and 3 % outside Europe. Almost half (49 %) of physicians had less than five years of medical experience and 29 % had recently graduated.

**Table 1:** Physicians’ personal and professional demographics

	Number	Percent
Physicians’ personal data		
Woman	34	(45)
Man	41	(55)
Physician with children	36	(48)
Physician without children	39	(52)
Country of birth - Norway	57	(76)
Country of birth – not Norway	18	(24)
Physicians’ education		
Country of study – Norway	55	(73)
Country of study – not Norway	20	(27)
Length of practice as physician		
< 5 years	37	(49)
≥ 5 years	38	(51)
Physicians’ main place of employment		
Accident and emergency department	52	(69)
General practice (GP)	21	(28)
Hospital	12	(16)

Table 2 shows the distribution of answers to questions about the physician’s management of pain in children. A total of 75 % of the physicians responded that they found it difficult to some degree to assess pain in children; 16 % found it hard or very hard, and only 9 % (*n* = 7) found it easy. There were no significant differences between the responses with regard to physicians’ personal and professional backgrounds.

**Table 2:** Physicians’ assessment of pain in children and adolescents (*n* = 75)

	Not at all		To a small extent		To some extent		Largely		To a very large extent	
	n	(%)	n	(%)	n	(%)	n	(%)	n	(%)
Do you often treat patients aged 0–19 years?	0	(0)	4	(5)	36	(48)	32	(43)	3	(4)
Do you find it difficult to assess pain in children?	0	(0)	7	(9)	56	(75)	9	(12)	3	(4)
Do you tend to use a pain scale for children aged 0 to 3 years?	51	(68)	22	(29)	1	(1)	1	(1)	0	(0)
Do you tend to use a pain scale for children aged 3 to 8 years?	29	(39)	29	(39)	11	(15)	6	(8)	0	(0)
Do you tend to use a pain scale for children and youth aged 8 to 19 years?	14	(19)	17	(23)	24	(32)	15	(20)	5	(7)
Do you tend to give medicine to your young patients based on age alone?	4	(5)	18	(24)	28	(37)	20	(27)	5	(7)
Do you tend to give medicine to your young patients based on weight alone?	0	(0)	0	(0)	8	(11)	44	(59)	23	(31)
Do you weigh your young patients yourself?	12	(16)	33	(44)	18	(24)	9	(12)	3	(4)
Do you tend to ask parents about the weight of your young patients?	0	(0)	0	(0)	16	(21)	37	(49)	22	(29)
Do you calculate the dose per kg body weight before giving painkillers to the child?	0	(0)	4	(5)	15	(20)	37	(49)	19	(25)
Do you follow Felleskatalogen’s dose recommendations in age/weight categories before giving painkillers to the child?^a^	0	(0)	0	(0)	7	(9)	40	(53)	27	(36)

^a^One physician did not answer

**Fig. 1:**
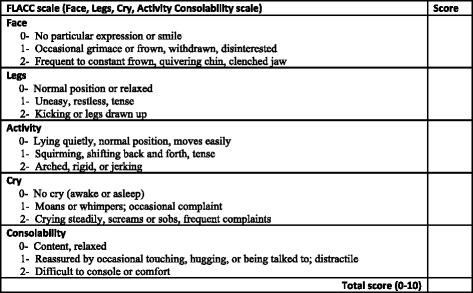
An observational pain intensity scale: The FLACC (Face Legs Activity Cry Consolability), extracted from Merkel SI [29]

**Fig. 2:**
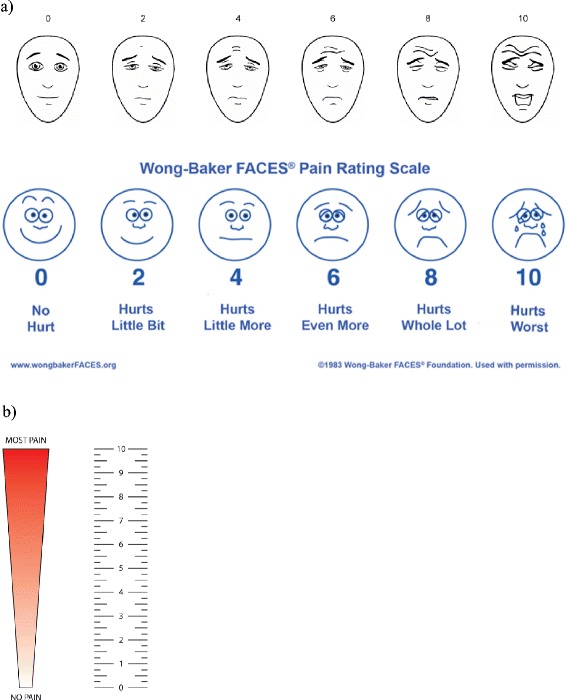
The three self-report pain scales in use for children >3 years and youth. **a** For children 4–12 years. FPS-R (Faces Pain Scale - Revised). Copyright 2001, International Association for the Study of Pain. Reproduced with permission from www.iasp-pain.org/FPSR. Wong-Baker FACES(R) Pain Rating Scale. Wong-Baker FACES Foundation (2015). Retrieved [01 Oct 2015] with permission from http://www.WongBakerFACES.org. [30]. **b** For children >8 years and youth. CAS (Colored Analogue Scale), adjusted from McGrath [31]

We asked if physicians used a pain scale for children and adolescents distributed in the three age groups <3 years, 3–8 years and 9–19 years (Figs. 1 and 2). Only 3 % of the physicians used a pain scale for children under three years of age; 23 % used it for children between 3 and 8 years, and 59 % for children and adolescents over 8 years of age. Physicians born in another European country used a pain scale for children under three years (*p* = 0.009) to a greater extent than other physicians. Physicians born outside Europe used a pain scale for children between 3 and 8 years (*p* = 0.01) to a greater extent than the others. Other professional or personal factors did not influence the use of a pain scale.

Most physicians answered that they took both weight and age into consideration when they dosed pain medication for children, while 5 % never took the child’s age into consideration when dosing painkillers. Weight alone was used to a significantly greater extent than age alone on drug dosing (*p* = 0.001. Most physicians (60 %) responded that they relied solely on parents’ estimation of their child’s weight when drugs were dosed. A total of 75 % of physicians calculated the dose of painkillers explicitly according to the child’s reported weight. 90 % responded that they mostly followed the recommendations of the Felleskatalogen in terms of age and weight groups. This practice was more common in physicians with less than five years of experience than the more experienced (*p* = 0.03).

A total of 93 % of physicians said, on a general basis, that to some or to a larger extent they wanted more knowledge about pain management in children [Table 3]. 88 % wanted to have a fixed procedure for assessment and 91 % wanted a fixed procedure for treatment of pain in children. Significantly more female physicians responded that they wanted more knowledge (*p* = 0.028), but otherwise, there were no significant differences with regard to physician’s personal and professional background.

**Table 3:** Physicians’ need for knowledge and procedures regarding treatment of pain in children and youth (*n* = 75)

	Not at all		To a small extent		To some extent		Largely		To a very large extent	
	n	(%)	n	(%)	n	(%)	n	(%)	n	(%)
Do you want more knowledge about pain management in children?	0	(0)	5	(7)	32	(43)	28	(37)	10	(13)
Do you want a fixed procedure for assessing pain in children?	0	(0)	9	(12)	26	(35)	30	(40)	10	(13)
Do you want a fixed procedure for treatment of pain in children?	0	(0)	7	(9)	29	(39)	30	(40)	9	(12)
Do you want other formulations than available analgesics to children in Norway?^a^	0	(0)	18	(24)	31	(41)	24	(32)	1	(1)
Overall, are you satisfied with your own handling of pain in children?	0	(0)	6	(8)	47	(63)	22	(29)	0	(0)

^a^One physician did not answer

Most physicians (75 %), and some to a very large extent, wanted alternative options to the available analgesics for children in Norway. Recently graduated physicians did not want other options as much as did the more experienced physicians (*p* = 0.009). There were no significant differences between physicians with and without children of their own.

A total of 63 % of the physicians said that they were satisfied to some extent with their own management of pain in children. Eight percent were little satisfied (five women, one man), while 29 % were largely satisfied (five women, 17 men). Significantly more female than male physicians were less satisfied with their own management of pain in children (*p* = 0.013). There were no significant differences between physicians with and without children, nor with regard to other personal or professional background.

## Discussion

This is the first Norwegian study to analyse how physicians themselves assess acute pain in young patients, and how they evaluate their own practice in this medical field.

In recent years, adequate pain relief has also been considered a human right [21]. Since pain treatment was stated as mandatory treatment in France in 2002, it is allowed for patients to sue a physician or hospital if customized pain relief is not given to a child or an adult [22]. Neither Norway nor other European countries have similar laws or law enforcements.

On a general basis, most physicians found it difficult to measure pain, but they were satisfied to some extent with their own management of pain. However, the large majority of physicians would like more knowledge and procedures with respect to both assessment and treatment of pain in children and youth from different diseases and injuries in a pre-hospital setting. A similar request for pain management procedures was mentioned in another study conducted in Norwegian hospitals in 2003 [7]. In our study, female physicians were less satisfied than their male colleagues with their own practice. They also reported wanting more knowledge and firm procedures than their male colleagues. A similar gender difference has previously been demonstrated among young physicians, and the difference may be explained by female physicians underestimating their levels of preparedness or tending to be more anxious and less professionally self-confident than their male colleagues [23].

According to different studies from hospital settings, not using a pain intensity scale or other pain scoring instrument may make the physicians’ evaluation of pain less consistent and more approximate than using a validated tool [5, 6, 9]. In trained physicians’ or nurses’ hands, self-report pain scales take less than one minute to use. Different well validated pain scales like Face, Legs, Activity, Cry, Consolability scale (FLACC scale), The Faces Pain Scale – Revised (FPS-R), Wong-Baker FACES® Pain Rating Scale (FACES), The Coloured Analogue Scale (CAS), visual analogue scale (VAS) (Figs. 1 and 2) and Numeric Rating Scale exist and are in use today and are adapted to different age levels [24, 25]. Unfortunately, lack of knowledge about how pain scales are used and the myth that it is time-consuming are obstacles to their use. Only 3 % of the physicians in our study answered that they used a pain scale for children under three years of age. The low rate among the youngest children may be explained by the hypothesis that many of the physicians neither knew about nor were able to define FLACC as a pain scale. Likewise, very few of our physicians had used pain scales in children aged three to 8 years, and were probably unaware that self-assessments can be done by some children from the age of three, and by most children from the age of five [12–14, 26]. More than half of the physicians in our study (59 %) had at some point used a pain scale for adolescents and for children older than 8 years. This may imply that physicians find pain scales useful, but they lack sufficient experience or knowledge to make use of it in younger and less communicative children. Anyway, the original purpose of pain scale was not to make an exact measurement of the pain intensity, but to make it possible to follow how pain develops with or without treatment over a period of time [9–11]. It is often argued that the busy pre-hospital situation at emergency and casualty centres demands quick and instant evaluations, and that the time aspect is an obstacle to make use of pain scales. However, many emergency procedures and even diagnostic evaluations depend on observations of a child over a longer or shorter period of time. Before repositioning a displaced fracture, and during observation of a child with fluctuating abdominal pain, the physician may find the use of a pain scale to be very valuable. Pain measurement should also be used as an essential component in evaluating the pain management of children in emergency primary care.

Almost all physicians in our study used the child’s weight rather than age in order to calculate the dose of painkillers. This is in accordance with pharmacological recommendations to avoid dosing errors if the child is above or below the normalized age dependent weight classes [17]. The majority of physicians only asked the parents about the child’s weight and did not request an additional weight control before prescribing medication to the child. Estimating instead of measuring weight can lead to dosage errors, and studies have shown that both parents and health professionals often underestimate the child’s weight [20]. With an increasing number of obese children in our western countries, dosage of medicine based on verified weight is important in order to provide effective treatment for many different conditions. Weighing the child should be implemented as a natural part of the routine procedures in the emergency primary care when the nurse welcomes the patient.

Nearly 90 % of the physicians follow the Felleskatalogen dosage recommendations to a very large extent. However, Felleskatalogen has limitations when it comes to drug dosing, especially to the youngest children weighing less than 10 kg. The pharmaceutical industry considers studies on drug use by the youngest children as risky, and few studies have been done [6]. Almost 90 % of medicine given to children in hospitals and about 50 % of the drugs given to children outside hospital are not scientifically approved [27]. Medication of our youngest children is often “off-labelled” use and implies either using known drugs for other indications, other doses of medication than specified from the producer or other forms of administration than specified in Felleskatalogen. As an example, it is proper treatment in acute pain or before initiating painful procedures to provide so-called greater saturation doses of analgesics [6, 17]. The analgesic doses can later be reduced so as not to exceed the maximum recommended doses during a certain time interval. Felleskatalogen does not account for such pre-hospital use, even though this is established as postoperative pain treatment of children in many hospitals [6]. Today, similar guidelines outside the hospital environment are not available in Norway.

Our study has several strengths and limitations. We had a high response rate among the emergency physicians. Still, the study included only 75 participants, which entails certain limitations in terms of interpreting the results, particularly when comparing the responses from sub-groups of physicians. There were almost as many female as male physicians included. This is also previously observed among physicians working in Norwegian casualty and pre-hospital emergency centres, but our physicians were on average two years younger (36.7 versus 39.1, p <0.05) [28]. This calls into scrutiny whether the answers from our physicians at Bergen AED are representative of physicians serving other emergency medical services in Norway. The average age of physicians working in the rural regions of Norway may be higher than physicians working closer to the university cities, where also recently graduated physicians make calls. The proportion of physicians in our study who were born abroad, was similar to the proportion in a representative sample of emergency medical physicians from other parts of Norway [28]. A key limitation of our study is that the data are based on self-report where recall bias may have taken place. We have no information about the physicians’ actual practice. Some answers may have been influenced by the fact that pain treatment of children had been in focus in our AED ahead of this study, and pain intensity scales for children had been demonstrated. Our questionnaire was not previously validated, and for this reason the physicians’ responses should also be interpreted with some caution.

## Conclusions

A relatively young, but otherwise representative sample of physicians participated in this first Norwegian study on pain assessment of children and youth in emergency primary care. Despite different personal and professional backgrounds, the physicians had surprisingly similar responses to questions about their own management of pain in young patients. A pain intensity scale was seldom used for children younger than eight years. A majority wanted more knowledge and procedures for both the evaluation and treatment of pain in children suffering from different medical and traumatic conditions. This study gives an impression that there is a large potential for improvement with regard to pre-hospital management of pain in children.

## Abbreviations

AED: Accident and emergency department
CAS: The coloured analogue scale
FACES: Wong-Baker FACES® pain rating scale
FLACC: Face, legs, activity, cry, consolability scale
FPS-R: The faces pain scale – revised
VAS: Visual analogue scale
